# The Effect of Smartphone Application–Based Self-Management Interventions Compared to Face-to-Face Diabetic Interventions for Pregnant Women With Gestational Diabetes Mellitus: A Meta-Analysis

**DOI:** 10.1155/jdr/4422330

**Published:** 2025-03-01

**Authors:** Thet Nu Khin, Wen Wei Ang, Ying Lau

**Affiliations:** ^1^Alice Lee Centre for Nursing Studies, Yong Loo Lin School of Medicine, National University of Singapore, Singapore; ^2^The Nethersole School of Nursing, The Chinese University of Hong Kong, Hong Kong Special Administrative Region, China

**Keywords:** face-to-face interventions, gestational diabetes mellitus, pregnant women, self-management, smartphone applications

## Abstract

**Background:** Face-to-face diabetic interventions (FFIs) are the gold standard for diabetic care, and smartphone application (app)–based self-management interventions (SBIs) can be a potential alternative. A few previous reviews compared the effects of both practices.

**Objectives:** This study is aimed at (1) comparing the effectiveness of FFIs and SBIs on maternal and neonatal outcomes in pregnant women with gestational diabetes mellitus (GDM) and (2) exploring potential covariates affecting those outcomes.

**Methods:** Randomized controlled trials (RCTs) were retrieved from PubMed, EMBASE, CINAHL, Cochrane Library, Scopus, and Web of Science from inception to January 15, 2024. Meta-analyses, subgroup analyses, and metaregression analyses were conducted using the *R* software package *meta*, Version 4.3.1. Cochrane risk of bias Version 2 (RoB2) and grading of recommendations, assessment, development, and evaluation (GRADE) criteria were employed to appraise the quality of studies and certainty of outcomes.

**Results:** We selected 15 RCTs from 2505 women with GDM across 11 countries for this review. The meta-analyses revealed that women in the SBIs can significantly reduce gestation weight gain (*t* = −2.45, *p* = 0.04) and macrosomia (*t* = −3.35, *p* = 0.02) when compared to those in the FFIs. We observed a higher likelihood of cesarean delivery when using generic apps (RR = 1.12, 95% confidence interval (CI): 0.59, 2.13) than GDM-specific apps (RR = 0.82, 95% CI: 0.64, 1.06). There was similar fasting plasma glucose, 2-h postprandial plasma glucose, hemoglobin A1c (HbA1c), cesarean section delivery rate, neonatal birthweight, large for gestational age, neonatal hypoglycemia, and neonatal intensive care unit admission between SBIs and FFIs. More than half (52%) were rated low risk based on RoB2. According to the GRADE criteria, very low to moderate evidence was found.

**Conclusions:** SBIs can be considered an alternative management method for women with GDM to reap the benefits of smartphone apps. More high-quality RCTs are required to reaffirm the findings.

## 1. Introduction

Gestational diabetes mellitus (GDM) was defined by O'Sullivan [1] as varied degrees of carbohydrate intolerance that first manifest or begin during pregnancy. According to a recent meta-analysis, 14.2% of people worldwide have GDM [2]. Because poor glycemic control can result in complications for both mothers and fetuses, the worldwide increase in GDM prevalence has become a global health concern [3]. This has led to a shortage of hospital resources for GDM education and management [4]. The lack of resources and the ever-increasing demand [5] will inevitably strain public healthcare systems. Given the worrying trends regarding the prevalence and its impacts, the development of innovative solutions can support the self-management of GDM and optimize glycemic control in pregnant women with GDM. Face-to-face diabetic interventions (FFIs) are the current standard care to monitor blood glucose levels and lifestyle factors through regular physical visits [6]. However, they also have limitations, including lack of accessibility due to waiting time incurred to consult healthcare professionals (HCPs), owing to a shortage of manpower in the healthcare system [7], and inconvenience in attending appointments [8], due to family and work responsibilities. Such barriers limit access to GDM care for a larger population, necessitating an alternative solution to tackle the current challenges posed by face-to-face GDM management interventions.

Smartphones [9] have gained popularity with the emergence and evolution of digitalization. Smartphones have their merits, such as portability, convenience of applications (apps), and constant internet connectivity, which can be utilized to address current challenges and become one of the treatment modalities for GDM. A systematic review has shown that mobile apps significantly improve self-efficacy, self-management behaviors, disease knowledge, therapeutic guidance, and patient-HCP communication [10]. Convenient transmission of blood glucose levels through mobile apps can enhance self-monitoring of blood glucose compliance, benefit HCPs by optimizing their time for education and monitoring, and enable them to collect more data related to glycemic control [4, 11]. Mobile apps can also offer two-way communication between patients and HCPs, reducing the burden of frequent appointments and improving communication [12]. Smartphones also host various social platforms, which can create opportunities for online peer support for GDM education and intervention. Women with GDM also seek informational and peer support and gain motivation to engage in behavioral change through social platforms [13]. With the widespread use of smartphones, harnessing their use provides a promising opportunity to improve the self-management of GDM.

Smartphone app–based self-management interventions (SBIs) may be an alternative approach to foster self-management behaviors through self-regulating cognitive processes according to social cognitive theory (SCT) [14]. Supporting Information 1 illustrates how SSIs can facilitate an increase in self-management behaviors among GDM patients. The SSI encourages self-management behaviors by improving knowledge of diabetes self-management, fostering self-efficacy, and enhancing problem-solving skills for successful adjustment and coping [15]. SCT also looks at how individual and environmental factors affect behavior, supporting the idea that social support and good communication between patients and HCPs can help diabetics stick to healthy habits [16, 17], which in turn lowers the risk of bad outcomes for both mothers and babies.

A growing body of reviews has demonstrated the potential benefits of technological advancements for pregnant women with GDM. However, some methodological limitations were found, such as a mixture of randomized controlled trials (RCTs) and non-RCTs [11, 18–20]; limited database searching [11, 19]; limited selected trials [9]; a mixture of all types of diabetes during pregnancy [21]; a broad population [18, 20]; a mixture of mobile phone text messages, wearable devices, apps, or other technologies [22, 23]; and solely narrative analysis [9, 18, 19]. None of the previous reviews conducted subgroup and metaregression analyses to investigate any potential covariates affecting the different outcomes. To fill the literature gap, our systematic review is aimed at (1) comparing the effectiveness of FFIs and SBIs on maternal and neonatal outcomes in pregnant women with GDM and (2) exploring potential covariates affecting those outcomes.

## 2. Methods

This systematic review and meta-analysis are reported based on the Preferred Reporting Items for Systematic Review and Meta-Analyses (PRISMA) checklist [24] (Supporting Information 2) as guidance in reporting this review. This review is registered in the study protocol in the International Prospective Register of Systematic Reviews (PROSPERO) with the number CRD42023403952.

### 2.1. Eligibility Criteria

A description of the eligibility criteria using the Population, Intervention, Comparator and Outcome (PICO) framework is provided in Supporting Information 3. This review included all types of RCTs consisting of pregnant women with GDM diagnoses. The interventions employed self-management strategies for GDM, utilizing smartphone apps that were based on the SCT framework. The features of SBIs can include any of the following components: data sharing, syncing, viewing; educational materials; patient-provider communication; and social support. Comparators were standard clinical care, which involved face-to-face scheduled appointments. Maternal outcomes include fasting plasma glucose (FPG), 2-h postprandial plasma glucose (2 h-PPG), hemoglobin A1c (HbA1c), gestational weight gain (GWG), and cesarean section (C-section) delivery. Neonatal outcomes include birth weight, incidence of macrosomia, large for gestational age (LGA), neonatal hypoglycemia, and neonatal intensive care unit (NICU) admission.

### 2.2. Search Strategy

A three-step search strategy was carried out to ensure extensive coverage [25]. A senior university librarian was consulted to ensure the search strategy was relevant and appropriate (Supporting Information 4). Six electronic databases were searched, such as PubMed, EMBASE (Ovid), CINAHL (EBSCOhost), Cochrane Library, Scopus, and Web of Science. Two reviewers also used the Peer Review of Electronic Search Strategies (PRESS) checklist [26] to guide them in assessing the search quality to minimize errors. Next, unpublished studies and ongoing trials were searched via grey literature, ClinicalTrials.gov, Google Scholar, and ProQuest Dissertations and Theses. After that, the reference lists of relevant articles were searched manually for additional studies. We did not impose any date restriction, but we only included articles in the English language. The search was completed from inception till January 15, 2024.

### 2.3. Data Extraction

The standardized data extraction form was used based on the *Cochrane Collaboration Handbook* [27]. Before the formal data extraction process, we first pilot-tested the form on six articles. The items extracted were the author's name, publication year, country, setting, study design, population, age, intervention, comparator, sample size, outcomes and measurement, drop-out rate, use of intention-to-treat analysis (ITT) and missing data management (MDM), availability of published protocol, and trial registration and grant support. Items extracted for features of interventions include diagnostic criteria of GDM, function, and components of the intervention, frequency, duration, and care provider. When the study publication did not provide the necessary additional information or when the reported data was insufficient for meta-analysis, authors of trials were contacted via email.

### 2.4. Quality Appraisal

The Cochrane risk of bias Version 2 (RoB2) [28] was used to judge and assess the quality of the involved RCTs. This tool's domains are (1) bias arising from the randomization process, (2) bias due to deviations from intended interventions, (3) bias due to missing outcome data, (4) bias in the measurement of the outcome, and (5) bias in the selection of the reported result. Two independent reviewers graded each item as either high risk, low risk, or some concerns. Drop-out rate, use of ITT/MDM, availability of published protocol, and trial registration were also considered when assessing the quality of the selected trials.

### 2.5. Certainty of Evidence and Publication Bias

The certainty of evidence was assessed using Grading of Recommendations, Assessment, Development, and Evaluation (GRADE) [29]. The summary of findings (SoFs) were developed using GRADEpro software [30], incorporating five categories for grading: risk of bias, inconsistency, indirectness, imprecision, and publication bias. The overall quality was categorized as very low, low, moderate, or high. When 10 or more trials have reported an outcome, we performed a funnel plot and Egger's test [31, 32] to assess publication bias. An asymmetrical funnel plot and a *p* value < 0.05 of Egger's test indicate publication bias [33].

### 2.6. Interrater Reliability

The selection of articles, data extraction, risk of bias appraisal, and GRADE criteria were assessed by two independent reviewers. Discussions resolved any differences in opinions, and they consulted a third reviewer if necessary. Cohen's kappa (*κ*) was calculated, and *κ* > 0.6 indicates adequate interrater reliability [34].

### 2.7. Meta-Analysis

Meta-analysis, subgroup, and metaregression were performed using the *meta* package in R software Version 4.3.1 [35]. An inverse variance method with a restricted maximum-likelihood estimator was used to get objective estimates [36]. The Hartung–Knapp adjustment for the random effects model was adopted to provide more adequate error rates [37]. We presented continuous outcomes as mean difference (MD) and dichotomous outcomes as risk ratio (RR), respectively, with a 95% confidence interval (CI). A 95% prediction interval (PI) was calculated to evaluate the potential effects for 95% of future similar trials [38]. If all 95% of the values fall on the same side of the null, there will be a statistically significant influence in the future [38]. If all values lie between the two sides of the null, there will not be any significant effect in the future [38].

Because most studies had small sample sizes, Hedges' *g* was used to measure the effect size [39]. The following were the parameters used to determine the effect size: very small (*g* = 0.01), small (*g* = 0.2), medium (*g* = 0.5), large (*g* = 0.8), very large (*g* = 1.2), and huge (*g* = 2.0) [40]. Heterogeneity within-study variability was evaluated using Cochran's *Q* statistic and the *I*^2^ index [41]. For Cochran's *Q*, a *p* value < 0.10 indicates that variation in effect estimates is not due to chance [27]. The *I*^2^ index was adopted to assess whether there is true heterogeneity, and the *I*^2^ value interprets heterogeneity as low if 0%–40%, moderate if 30%–60%, substantial if 50%–90%, and considerable if 75%–100% [27].

### 2.8. Subgroup and Metaregression Analyses

Subgroup analysis and metaregression were performed for an outcome in which at least 10 studies have reported identifying the underlying issue of heterogeneity further [27]. Subgroup analyses were done for categorical covariates [42]. The predetermined subgroups included region (Asian vs. non-Asian), type of apps (generic apps vs. GDM-specific apps), and type of GDM diagnostic criteria (International Association of Diabetes and Pregnancy Study Group criteria (IADPSG) [43] endorsed by the World Health Organization (WHO) [44], the International Federation of Gynecology and Obstetrics (FIGO) criteria [45], or the Carpenter–Coustan criteria [46]). If the *p* value > 0.1, the subgroup effect is statistically significant [47]. Metaregression analyses were conducted for continuous covariates like publication year, mean age, attrition rate, and sample size [27]. The significance level is set at *p* < 0.05, and the regression coefficient (*β*) was reported to show the estimated change in the outcome variable for every unit increase of a covariate [27].

## 3. Results

### 3.1. Selection of Studies

**Figure 1:**
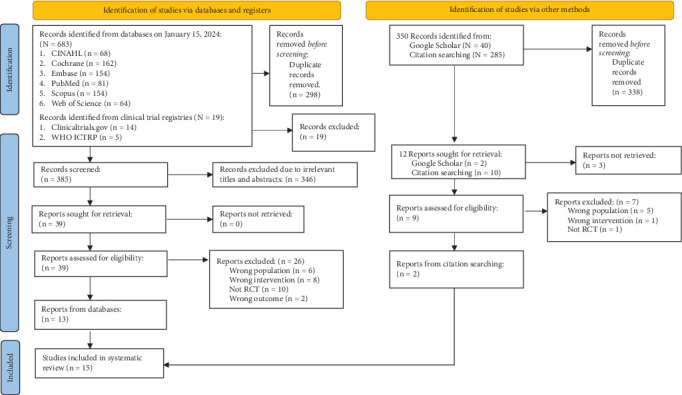
PRISMA flow diagram.

The PRISMA flow diagram (Figure 1) illustrates the steps to select articles [24]. A total of 683 articles from the preliminary search were imported using the search strategy into the EndNote X20 software program [48]. After removing 298 duplicates, 346 out of 385 screened articles were omitted due to their unfit titles and abstracts. We retrieved the remaining 39 articles and assessed their eligibility. Supporting Information 5 notes the reasons for the exclusion of 26 full-text records. Supporting Information 6 and 7 record the reasons for other excluded studies. Eventually, 15 RCTs were included [49–63] after adding two from other sources.

### 3.2. Characteristics of Included RCTs

The details of selected RCTs are outlined in Table 1. All articles were two-armed RCTs involving 2505 pregnant women with GDM. The diagnostic criteria of GDM were used according to IADPSG [43] endorsed by the WHO [44] (*n* = 6), the ADA criteria [64] (*n* = 5), the FIGO criteria [45] (*n* = 1), and the Carpenter-Coustan criteria [46] (*n* = 1). However, two studies [49, 50] did not specify the criteria used. The studies were conducted across nine countries: Saudi Arabia [51] (*n* = 1), Norway [49] (*n* = 1), the United Kingdom [52] (*n* = 1), Iran [53] (*n* = 1), Israel [54] (*n* = 1), Slovenia [55] (*n* = 1), Spain [56] (*n* = 1), Turkey [57] (*n* = 1), Singapore [58] (*n* = 1), South Korea [50] (*n* = 1), and China [59–63] (*n* = 5). More than half of the studies (*n* = 8) came from higher income countries (50–53, 55–57, and 59), and seven studies came from upper-income countries (54, 58, and 60–64). Most of the studies (73.33%) were conducted in hospitals, while two were done in outpatient clinics, one at a medical center, and one at a public health center. The number of participants varied from 21 to 400.

### 3.3. Description of Smartphone App–Based Interventions

Supporting Information 8 records the description of SBIs among the included trials. Most of the interventions are specific smartphone apps developed for GDM, except for three studies [60, 61, 63], which utilized existing social media platforms (i.e., the WeChat app) to deliver the intervention. The frequency of the interventions varies across studies, and the duration of studies was not documented explicitly in the articles, except for the study by Simsek-Cetinkaya and Koc [57], which lasted for 14 weeks. Supporting Information 9 categorizes the components of the interventions into four types. Eleven studies included the features of data sharing, syncing, or viewing. All the studies included educational materials, either in real-time or embedded as part of the app. Ten studies involved patient–provider communication. Only three studies [57, 60, 61] included social support features.

### 3.4. RoB2

Seven trials used ITT, and eight trials used per-protocol analysis (Supporting Information 10). Only three studies (20%) had a low risk of bias in the randomization process domain. Most studies failed to disclose whether they concealed the allocation sequence. Due to the nature of interventions, all included studies expressed some concerns about deviations from the intended interventions domain. Only four studies had some concerns about the missing outcome data domain. All selected studies had a low risk of bias in measuring the outcome, and one-third of them had a low risk of bias in selecting the reported result. More than half (52%) were low-risk across five domains.

### 3.5. Maternal Outcomes

**Figure 2:**
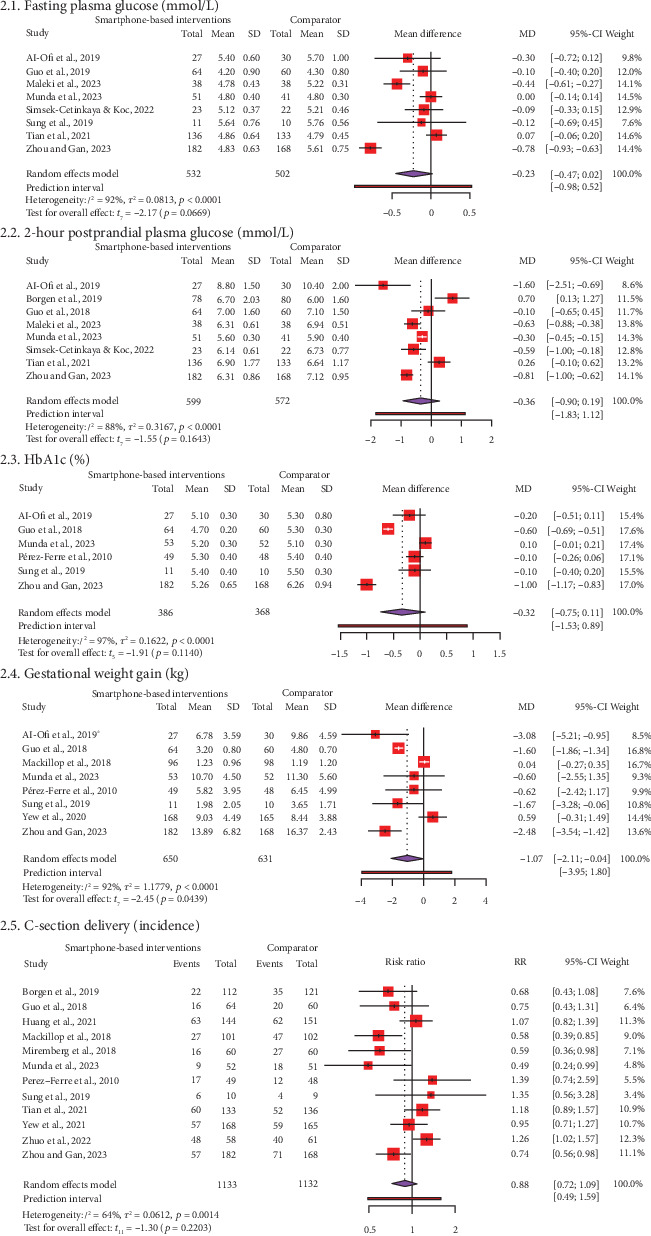
Forest plot of mean difference/risk ratio for maternal outcomes between smartphone application–based self-management intervention and face-to-face diabetic intervention groups.

Figure 2 and Supporting Information 11 display forest plots of MD or RR and effect sizes (Hedge's *g*) for maternal outcomes, including FPG among 1034 women in eight RCTs, 2 h-PPG among 1171 women in eight RCTs, HbA1c among 754 women in six RCTs, GWG among 1281 women in eight RCTs, and C-section among 2265 women in 12 RCTs. Meta-analysis showed that SBIs can significantly reduce 1.07 kg compared to FFIs (95% CI: −2.11, −0.04, *t* = −2.45, *p* = 0.04) with a median effect size (*g* = −0.69, 95% CI: −1.71, 0.32). Heterogeneity was considerably high (*I*^2^ = 92%, *p* < 0.01). The PI varied from −3.95 to 1.80, with values obtained on both sides of the null (0), indicating that the SMIs did not substantially decrease GWG when compared to FFIs in comparable future research. However, there was no significant statistical difference between SBIs and FFIs for FPG (MD = −0.23, 95% CI: −047, 0.02, *t* = −2.17, *p* = 0.07), 2 h-PPG (MD = −0.36, 95% CI: −0.90, 0.19, *t* = −1.55, *p* = 0.16), HbA1c (MD = −0.32, 95% CI: −0.75, 0.11, *t* = −1.91, *p* = 0.11), and C-section (RR = 0.88, 95% CI: 0.72, 1.09, *t* = −1.03, *p* = 0.22). The effect sizes ranged from small to median (*g* = −0.39 to −0.69). Substantial to considerable heterogeneities were observed (*I*2 = 64%–97%).

Subgroup and metaregression analyses were conducted because 12 studies reported on C-section delivery. Subgroup analyses were done according to region, types of apps, and GDM diagnostic criteria (Table 2). A significant subgroup difference between the types of apps (*p* = 0.01) was detected. We observed a higher likelihood of C-section delivery when using generic apps (RR = 1.12, 95% CI: 0.59, 2.13) than GDM-specific app*s* (RR = 0.82, 95% CI: 0.64, 1.06). There were similar RRs across the regions and GDM criteria subgroups. However, the reason for heterogeneity remained unexplained. Furthermore, a series of metaregression analyses (Table 3) was conducted to determine whether any continuous covariates influence the effect size of C-section delivery. All the covariates did not affect the incidence of C-section delivery, such as sample size (*β* ≤ −0.01, *p* = 0.73), attrition rate (*β* = 0.01, *p* = 0.59), and year of publication (*β* = −0.01, *p* = 0.74).

### 3.6. Neonatal Outcomes

**Figure 3:**
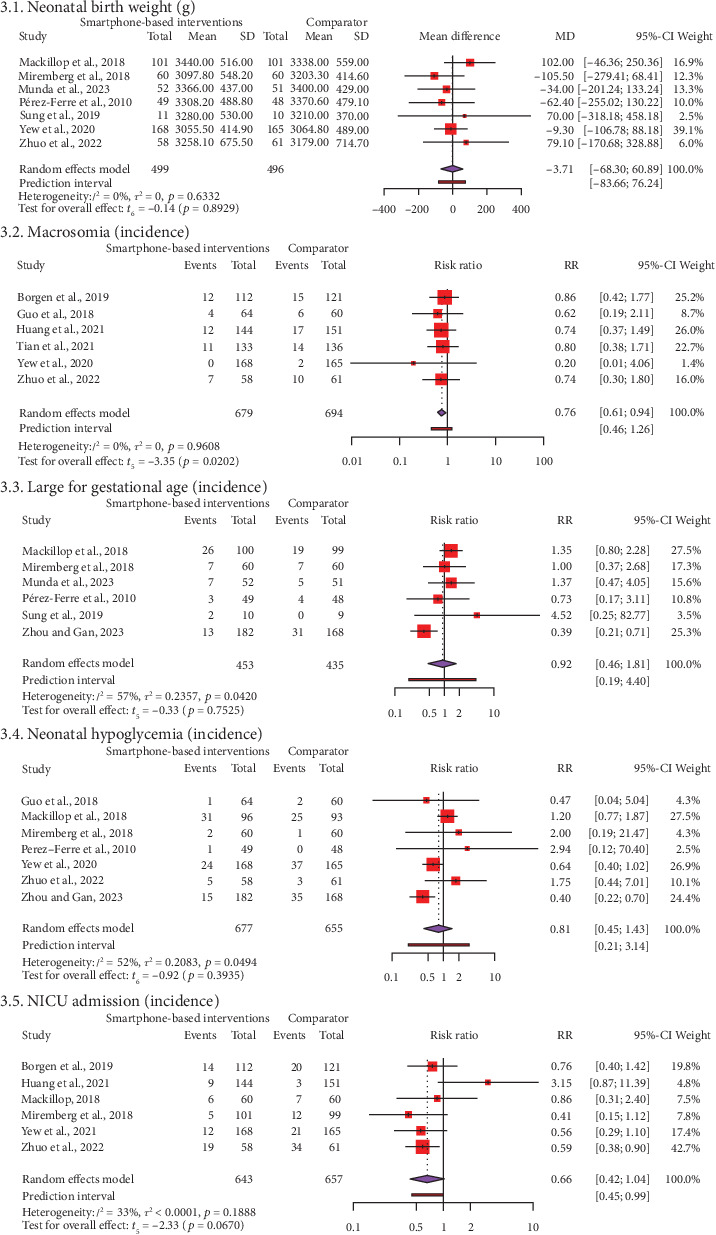
Forest plot of mean difference/risk ratio for neonatal outcomes between smartphone application–based self-management intervention and face-to-face diabetic intervention groups.

Figure 3 and Supporting Information 12 show the results of our study on neonatal outcomes. These included birth weight for 995 neonates in seven RCTs, the incidence of macrosomia for 1373 neonates in 6 RCTs, the incidence of LGA for 888 neonates in 6 RCTs, the incidence of hypoglycemia for 1332 neonates in seven RCTs, and NICU admissions for 1300 neonates in six RCTs. Our meta-analysis revealed that the incidence of macrosomia was significantly lower in SBIs than in FFIs (RR = 0.76, 95% CI: 0.61, 0.94, *t* = −3.35, *p* = 0.02). The PI ranged from 0.46 to 1.26, with values on both sides of the null (1). This suggests that SMIs did not significantly lower the incidence of macrosomia when compared to FFIs in future studies of the same kind. Meta-analyses show that using SBIs was not statistically different from using FFIs for birthweight (MD = −3.71, 95% CI: −68.30, 60.89, *t* = 0.14, *p* = 0.89), LGA (RR = 0.92, 95% CI: 0.46, 1.81, *t* = −0.33, *p* = 0.75), hypoglycemia (RR = 0.81, 95% CI: 0.45, 1.43, *t* = −0.92, *p* = 0.39), and NICU admission (RR = 0.66, 95% CI: 0.42, 1.04, *t* = −2.33, *p* = 0.07). We found no heterogeneity and sustainable heterogeneities (*I*^2^ = 0%–57%) for all neonatal outcomes.

### 3.7. Certainty of Evidence and Publication Bias

The certainty of the evidence was evaluated using the GRADE criteria (Supporting Information 13). All outcomes scored very low to moderate. Methodological limitations, high heterogeneities, and variability in interventions downgraded the domains of risk of bias, inconsistency, and indirectness. A visual examination of the funnel plot reveals the symmetrical distribution of 12 trials on C-section delivery (Supporting Information 14). Egger's test (*p* = 0.154) further confirms this, revealing no publication bias. The acceptable interrater reliability results between two independent reviewers were found: *κ* = 0.78 for the selection of articles, *κ* = 0.86 for the data extraction, *κ* = 0.82 for the risk of bias appraisal, and *κ* = 0.88 for the GRADE criteria.

## 4. Discussion

We included 15 RCTs with a total of 2505 women with GDM from 11 countries to compare the effectiveness of SBIs and FFIs on maternal (FPG, 2 h-PPG, HbA1c, GWG, and C-section rate) and neonatal outcomes (birthweight, macrosomia, LGA, hypoglycemia, and NICU admission). Our meta-analyses revealed that SBIs significantly reduce the GWG and incidence of macrosomia when compared to FFIs. Subgroup analysis showed women using generic apps had a higher likelihood of C-section delivery than women using GDM-specific apps. Metaregression analyses found that sample size, attrition rate, and year of publication did not affect the incidence of C-section delivery. The results of our meta-analyses showed that the effects of SBIs and FFIs on maternal (FPG, 2 h-PPG, HbA1c, and C-section rate) and newborn (birthweight, LGA, hypoglycemia, and NICU admission) outcomes are comparable. Across five domains, almost half (52%) were low-risk based on RoB2. According to the GRADE criteria, the evidence's certainty ranged from very low to moderate for all outcomes. No publication bias was observed.

This review shows that GDM women in the SBIs group can significantly reduce their GWG and incidence of macrosomia compared to GDM women in the FFIs group. The results echoed previous reviews [11, 21] that showed remote monitoring technologies decreased GWG, and telemedicine in managing diabetes in pregnancy reduced the risk of shoulder dystocia. Indeed, reports consistently show that the incidence of shoulder dystocia rises with macrosomia [65]. One possible reason to explain this result is that SBIs encourage self-regulation of daily physical activities, healthy diets, and weight management according to the SCT framework [14].

Furthermore, the smartphone app also provides social support to improve self-management behavior [15]. One qualitative study [12] also showed that GDM women viewed smartphones as convenient, beneficial, and trustworthy, and they valued mobile health intervention for seeking information, peer support, and lifestyle change. This may have caused a significant reduction in GWG and macrosomia. This implies that SBIs can maintain healthy weight gain and reduce the incidence of macrosomia and are better than FFIs.

This review demonstrated that there is no significant disparity in glycemic control between SBIs and FFIs, as evidenced by the insignificant difference in FPG, 2 h-PPG, and HbA1c. This is supported by a previous meta-analysis [23], which showed that mobile interventions are not significantly different from usual care on FPG, 2 h-PPG, and HbA1c of GDM patients through the subgroup analysis conducted among other digital interventions. Nonetheless, the seamless transmission of blood glucose values from the glucometer to the apps was able to provide convenience to GDM patients. A previous study [6] found that some of the barriers to improving GDM care are a lack of reminders on blood glucose monitoring and a lack of communication with HCPs to seek advice regarding their glycemic control. One qualitative study also suggested a tailored approach can improve engagement and adaptation to mobile-based interventions [12]. SBIs were able to provide equally effective glycemic control as FFIs, with less involvement from HCPs. SBIs can streamline and personalize GDM care, making them viable alternatives for optimizing glycemic control among GDM patients.

This review revealed that there is a similar C-section delivery rate between SBIs and FFIs. However, the current literature showed mixed results on the C-section delivery rate. A meta-analysis review [66] established that there is no difference in the C-section delivery rate with technology-supported lifestyle interventions compared to usual care. On the other hand, a meta-analysis [67] discovered that telemedicine interventions have a significant impact on decreasing the incidence of C-section delivery rates. However, it is notable that some studies did not report on the planned and emergency C-sections separately. This may have caused discrepancies in the analysis, as planned C-sections may have been due to mothers' personal choice rather than being considered as an adverse outcome of uncontrolled GDM. Our review included five studies [59–63] in China, where a high C-section rate of 45.2% was found to be attributed to a multitude of social factors in China, including maternal requests [68]. Furthermore, our subgroup analysis observed a higher likelihood of C-section delivery when using generic apps than GDM-specific apps. The GDM-specific app may provide women with more specialized features to enhance GDM management compliance, potentially reducing complications [9]. As a result, the C-section delivery rates decreased. Nevertheless, we can conclude that using SBIs instead of FFIs does not cause a significant increase in the C-section delivery rate and even has the potential to lower the rate with the inclusion of more studies using the GDM-specific app.

Our reviews showed that similar neonatal birth weight, LGA, incidence of neonatal hypoglycemia, and NICU admission were found between SBIs and FFIs. Similarly, these results support earlier meta-analyses [23, 66, 69] that showed technologically supported interventions did not have adverse effects on neonatal outcomes such as birthweight, LGA, hypoglycemia, and NICU admission, even though SBIs needed less manpower and time to manage GDM than FFIs.

Our review has several benefits. First, with meticulous, comprehensive, and methodical searches and analyses, this study was able to reduce bias and overcome the methodological shortcomings of previous comparable reviews. Second, the source of heterogeneity was examined using subgroup and metaregression analysis. Third, a random effects meta-analysis using the Hartung–Knapp adjustment was carried out to prevent paradoxical effects [37]. Fourth, to forecast the result of an upcoming similar study, 95% of PI was also provided [38].

There are several limitations in this systematic review. First, the review was limited to English literature, which restricted the findings' generalizability. Second, most of the studies did not standardize the gestational age at recruitment, which would have had an impact on the variation in intervention length. Third, the inclusion of different types of mobile apps in this review may have led to clinical heterogeneity among the studies. Fourth, we found high heterogeneities for some outcomes, suggesting an incorrect calculation of the effect. Finally, for some outcomes, the certainty of evidence was either very low or low, which may undermine the internal validity of the results.

The lack of apps specifically designed for the GDM population demonstrates the underutilization of SBIs in current clinical practice. Based on the evidence presented in this review, SBIs can significantly reduce GWG and macrosomia incidence. SBIs did not negatively affect maternal and neonatal outcomes. Given their widespread use in this age, smartphones can be a medium for the delivery of low-cost, user-friendly, and personalized interventions. The SBIs can also improve patients' satisfaction and, possibly, compliance. Even among developing countries, where there is a shortage of HCPs, access to mobile phones is high [70]. As a result, SBIs can be considered an adjunct to usual care and could be a long-term solution for better GDM care provision.

However, more high-quality RCTs are required to increase the overall confidence in the effect estimation. There are also very few studies that report on compliance rates, and when they do, there is a lack of standardization in reporting. Therefore, we recommend that future research use a standardized definition of compliance and report these data. Given the diverse number of GDM diagnostic criteria available, we recommend that future RCTs use standardized criteria to recruit participants. We can also conduct qualitative studies to gain insight into the experience of using smartphone apps in GDM management. Future research can also focus on the cost-effectiveness of SBIs to determine whether they are a better alternative to usual care in the long run.

## 5. Conclusion

This review highlights that women in the SBIs can significantly reduce the GWG and incidence of macrosomia when compared to the FFIs. The SBIs can deliver as effective GDM care as FFIs, without compromising on maternal (FPG, 2 h-PPG, HbA1c, and C-section delivery) and neonatal outcomes (birthweight, LGA, hypoglycemia, and NICU admission). As a result, SBIs could be considered an adjunct intervention to usual care, providing well-rounded support for GDM patients while easing the healthcare burden. We should address the limitations in this review and include more high-quality RCTs to reaffirm the findings.

## Figures and Tables

**Table 1:** Characteristics of the included trials (*N* = 15).

**Author, year [reference]**	**Country/income/setting**	**Design**	**Population**	**Age**	**Smartphone application–based self-management interventions (name)**	**Comparator**	**Sample size**	**Maternal and neonatal outcome**	**Attrition rate (%)**	**ITT/MDM**	**Protocol/registry**	**Grant**
Al-ofi et al., 2018 [51]	Saudi Arabia/high hospital	2-arm RCT	GDM	I: 32.5 ± 5.8 C: 32.4 ± 5.3	Smartphone-glucometer and a Glucomail application to monitor blood glucose and weight	Face-to-face diabetic intervention	57	- FPG - 2 h-PPG - HbA1c - GWG	5	No/no	No/no	Yes

Sung et al., 2019 [50]	Norway/high/clinic	2-arm RCT	GDM diagnosed, < 33 weeks of gestation	≥ 18	Smartphone application for GDM management (Pregnant+ app)	Face-to-face diabetic intervention	233	- 2 h-PPG - C-section delivery - Macrosomia - NICU admission	2	Yes/no	Yes/yes	Yes

Guo et al., 2018 [59]	China/upper-middle/hospital	2-arm RCT	GDM diagnosis	I: 31.2 ± 4.1 C: 30.6 ± 3.1	Mobile health app for GDM management (Dnurse)	Face-to-face diabetic intervention	124	- FPG - 2 h-PPG - HbA1c - GWG - C-section delivery - Macrosomia - Neonatal hypoglycaemia	0	No/no	No/no	Yes

Huang et al., 2021 [60]	China/upper-middle/hospital	2-arm RCT	GDM diagnosed	30.95 ± 4.45	Social media app for self-management of diet, exercise, and glucose control (WeChat)	Face-to-face diabetic intervention	295	- C-section delivery - Macrosomia - NICU admission	12	No/no	No/no	Yes

Mackillop et al., 2018 [52]	United Kingdom/high/hospital	2-arm RCT	GDM diagnosed, < 35 weeks of gestation	I: 33.9 ± 5.5 C: 33.0 ± 5.6	Mobile phone–based blood glucose management (GDm-health)	Face-to-face diabetic intervention	203	- GWG - C-section delivery - Neonatal birth weight - LGA - Neonatal hypoglycaemia - NICU admission	1	Yes/yes	Yes/yes	Yes

Maleki et al., 2023 [53]	Iran/upper-middle/public health center	2-arm RCT	GDM diagnosed, 24–28 weeks of gestation	I: 30.47 ± 3.13 C: 28.92 ± 2.44	Mobile-assisted education on health promoting lifestyle and blood sugar of women with gestational diabetes	Face-to-face diabetic intervention	76	- FPG - 2 h-PPG	0	No/no	Yes/yes	Yes

Miremberg et al., 2018 [54]	Israel/high/medical centre	2-arm RCT	GDM diagnosed, < 34 weeks of gestation	I: 31.7 ± 4.2 C: 32 ± 6.3	Smartphone application (GlucoseBuddy)	Face-to-face diabetic intervention	120	- C-section delivery - Neonatal birth weight - LGA - Neonatal hypoglycaemia - NICU admission	5	No/no	No/yes	No

Munda et al., 2023 [55]	Slovenia/high/clinic	2-arm RCT	GDM diagnosed, < 30 weeks of gestation	I: 32.8 C: 32	Telemedicine intervention for GDM, sending glucose readings via an application installed on a smartphone	Face-to-face diabetic intervention	106	- FPG - 2 h-PPG - HbA1c - GWG - C-section delivery - Neonatal birth weight - LGA	7	Yes/no	No/yes	No

Perez-Ferre et al., 2010 [56]	Spain/high/hospital	2-arm RCT	GDM diagnosed	I: 33.33 ± 5.58 C: 34.19 ± 5.18	Mobile phone application for the transmission of SMBG values to the central database through SMS	Face-to-face diabetic intervention	97	- HbA1c - GWG - C-section delivery - Neonatal birth weight - LGA - Neonatal hypoglycaemia	3	No/no	No/yes	Yes

Simsek-Cetinkaya and Koc, 2022 [57]	Turkey/upper-middle/hospital	2-arm RCT	GDM	≥ 18	Smartphone-based nursing counselling and feedback app	Face-to-face diabetic intervention	45	- FPG - 2 h-PPG	10	No/no	No/no	No

Sung et al., 2019 [50]	South Korea/high/hospital	2-arm RCT	GDM diagnosed, ≤ 30 weeks of gestation	I: 35 ± 2.76 C: 31.7 ± 4.92	Mobile application for GDM self-management (Huraypositive Inc)	Face-to-face diabetic intervention	21	- FPG - HbA1c - GWG - Neonatal birth weight - LGA	0	Yes/no	No/yes	Yes

Tian et al., 2021 [61]	China/upper-middle/hospital	2-arm RCT	GDM diagnosed, < 31 weeks of gestation	I: 31.23 ± 4.21 C: 30.93 ± 4.48	Social media app blood glucose management (WeChat)	Face-to-face diabetic intervention	269	- FPG - 2 h-PPG - C-section delivery - Macrosomia	13	No/no	No/yes	Yes

Yew et al., 2021 [58]	Singapore/high/hospital	2-arm RCT	GDM diagnosed, 12–30 weeks of gestation	I: 31.7 ± 4.0 C: 32.2 ± 4.4	Smartphone app coaching program (Habits-GDM)	Face-to-face diabetic intervention	340	- GWG - C-section delivery - Neonatal birth weight - Macrosomia - Neonatal hypoglycaemia - NICU admission	2	Yes/no	No/yes	Yes

Zhou and Gan, 2023 [63]	China/upper-middle/hospital	2-arm RCT	GDM diagnosed, < 28 weeks of gestation	I: 30.27 ± 3.21 C: 29.89 ± 3.42	Personalized education and supervision (WeChat)	Face-to-face diabetic intervention	400	- FPG - 2 h-PPG - HbA1c - GWG - C-section delivery - LGA - Neonatal hypoglycaemia	12.5	No/no	No/no	Yes

Zhuo et al., 2022 [62]	China/upper-middle/hospital	2-arm RCT	GDM diagnosed, 24–35 weeks of gestation	I: 31.8 ± 5.2 C: 32.7 ± 5.9	Smartphone app for diabetes self-management (continuing medical care)	Face-to-face diabetic intervention	119	- C-section delivery - Neonatal birth weight - Macrosomia - Neonatal hypoglycaemia - NICU admission	4	Yes/no	No/no	Yes

*Note:* Income, classification of countries based on the World Bank (2024); High, high-income country according to the Work Bank (2024); Upper-middle, upper-middle country according to the Work Bank (2024). Reference: World Bank (2024). World Development Indicators. Retrieved from https://www.worldbank.org/ext/en/home.

Abbreviations: 2 h-PPG, 2-h postprandial plasma glucose; App, application; C, control; C-section delivery, cesarean section delivery; FPG, fasting plasma glucose; GDM, gestational diabetes mellitus; GWG, gestation weight gain; I, intervention; ITT, intention-to-treat analysis; LGA, large for gestational age; MDM, missing data management; NR, not reported; RCT, randomized controlled trial; SMS, short message service.

**Table 2:** Subgroup analysis of categorical covariates on cesarean section delivery.

	**Subgroups**	**No. of trials [References, authors, year]**	**Sample size**	**I** ^2^	**Effect size**	**RR, 95% CI**	**Subgroup differences**
Region	Asian	7 [Guo et al., 2018; Huang et al., 2021; Sung et al., 2019; Tian et al., 2021; Yew et al., 2021; Zhuo et al., 2021; Zhou and Gan, 2023]	1417	69%	*t* = −0.67 *p* = 0.53	RR: 0.93 95% CI: 0.70, 1.23	*χ* ^2^ = 0.41 *p* = 0.52
	Non-Asian	5 [Borgen et al., 2019; Mackillop et al., 2018; Miremberg et al., 2018; Munda et al., 2023; Perez-Ferre et al., 2010]	848	61%	*t* = −1.19 *p* = 0.30	RR: 0.81 95% CI: 0.49, 1.33	

Types of applications	Generic apps	2 [Huang et al., 2021; Tian et al., 2021]	564	0%	*t* = 2.18 *p* = 0.27	RR: 1.12 95% CI: 0.59, 2.13	*χ* ^2^ = 6.34 *p* = 0.01^∗^
	GDM-specific apps	10 [Borgen et al., 2019; Guo et al., 2018; Mackillop et al., 2018; Miremberg et al., 2018; Munda et al., 2023; Perez-Ferre et al., 2010; Sung et al., 2019; Yew et al., 2021; Zhuo et al., 2021; Zhou and Gan, 2023]	1701	66%	*t* = −1.76 *p* = 0.11	RR: 0.82 95% CI: 0.64, 1.06	

GDM diagnostic criteria	IADPSG/WHO	4 [Mackillop et al., 2018; Munda et al., 2023; Yew et al., 2021; Tian et al., 2021]	908	74%	*t* = −1.12 *p* = 0.34	RR: 0.80 95% CI: 0.43, 1.50	*χ* ^2^ = 1.15 *p* = 0.56
	ADA	4 [Guo et al., 2018; Miremberg et al., 2018; Zhuo et al., 2021; Zhou and Gan, 2023]	713	78%	*t* = −1.05 *p* = 0.37	RR: 0.84 95% CI: 0.49, 1.43	
	Nonspecific criteria/NR	4 [Borgen et al., 2019; Huang et al., 2021; Perez-Ferre et al., 2010; Sung et al., 2019]	694	31%	*t* = −0.09 *p* = 0.93	RR: 1.01 95% CI: 0.63, 1.64	

*Note:I* ^2^, heterogeneity (proportion of total variation across studies that is due to heterogeneity between studies rather than by chance); *Q*, Cochran *Q* statistic (result from heterogeneity beyond chance).

Abbreviations: ADA, American Diabetes Association; Apps, applications; GDM, gestational diabetes mellitus; IADPSG, International Association of the Diabetes and Pregnancy Study Groups; NR, not reported; RR, risk ratio; WHO, World Health Organization.

⁣^∗^Statistically significant for subgroup analysis (*p* < 0.1).

**Table 3:** Random-effects metaregression of continuous covariates on cesarean section delivery.

**Covariates**	**β**	**SE**	**95% CI**	**p** ** value**
Sample size	< −0.01	< 0.01	< −0.01, < 0.01	0.73
Attrition rate	0.01	0.02	−0.03, 0.06	0.59
Year of publication	−0.01	0.03	−0.09, 0.06	0.74

*Note:β*: coefficient.

Abbreviations: CI, confidence interval; SE, standard error.

## Data Availability

The original contributions presented in the study are included in the article/supporting information, and further inquiries can be directed to the corresponding author.

## Supporting Information

Supporting Information 1 Potential mechanisms of smartphone application–based self-management intervention on maternal and neonatal outcomes among pregnant women with GDM.

Supporting Information 2 PRISMA 2020 checklist.

Supporting Information 3 Eligibility criteria.

Supporting Information 4 Search terms in databases.

Supporting Information 5 Excluded full-text articles with reasons (*n* = 26).

Supporting Information 6 Exclusion of trials from registries with reasons (*n* = 19).

Supporting Information 7 Excluded articles from other sources with reasons (*n* = 10).

Supporting Information 8 Description of smartphone-based self-management interventions among included studies.

Supporting Information 9 Features of smartphone application–based self-management interventions.

Supporting Information 10 Risk of bias Version 2 summary.

Supporting Information 11 Forest plot of effect sizes (Hedges' *g*) for maternal outcomes between smartphone application–based self-management intervention and face-to-face intervention groups.

Supporting Information 12 Forest plot of effect size (Hedges' *g*) for neonatal birthweight between smartphone application–based self-management intervention and face-to-face intervention groups.

Supporting Information 13 GRADE assessment for maternal and neonatal outcomes.

Supporting Information 14 Funnel plot showing symmetrical distribution and Egger's test of 12 trials for caesarian delivery.
