# Supplementary material for: The Effect of Smartphone Application–Based Self-Management Interventions Compared to Face-to-Face Diabetic Interventions for Pregnant Women With Gestational Diabetes Mellitus: A Meta-Analysis
Source: J Diabetes Res. 2025 Mar 1;2025:4422330. doi: 10.1155/jdr/4422330 (PMC11986943; doi:10.1155/jdr/4422330)
Supplement: Supporting Information 1 — Potential mechanisms of smartphone application–based self-management intervention on maternal and neonatal outcomes among pregnant women with GDM. [file 4422330.f1.docx]

**The effect of smartphone application-based self-management interventions compared to face-to-face diabetic interventions for pregnant women with gestational diabetes mellitus: A meta-analysis**

Supporting Information 1: Potential mechanisms of smartphone application-based self-management intervention on maternal and neonatal outcomes among pregnant women with gestational diabetes mellitus.

**
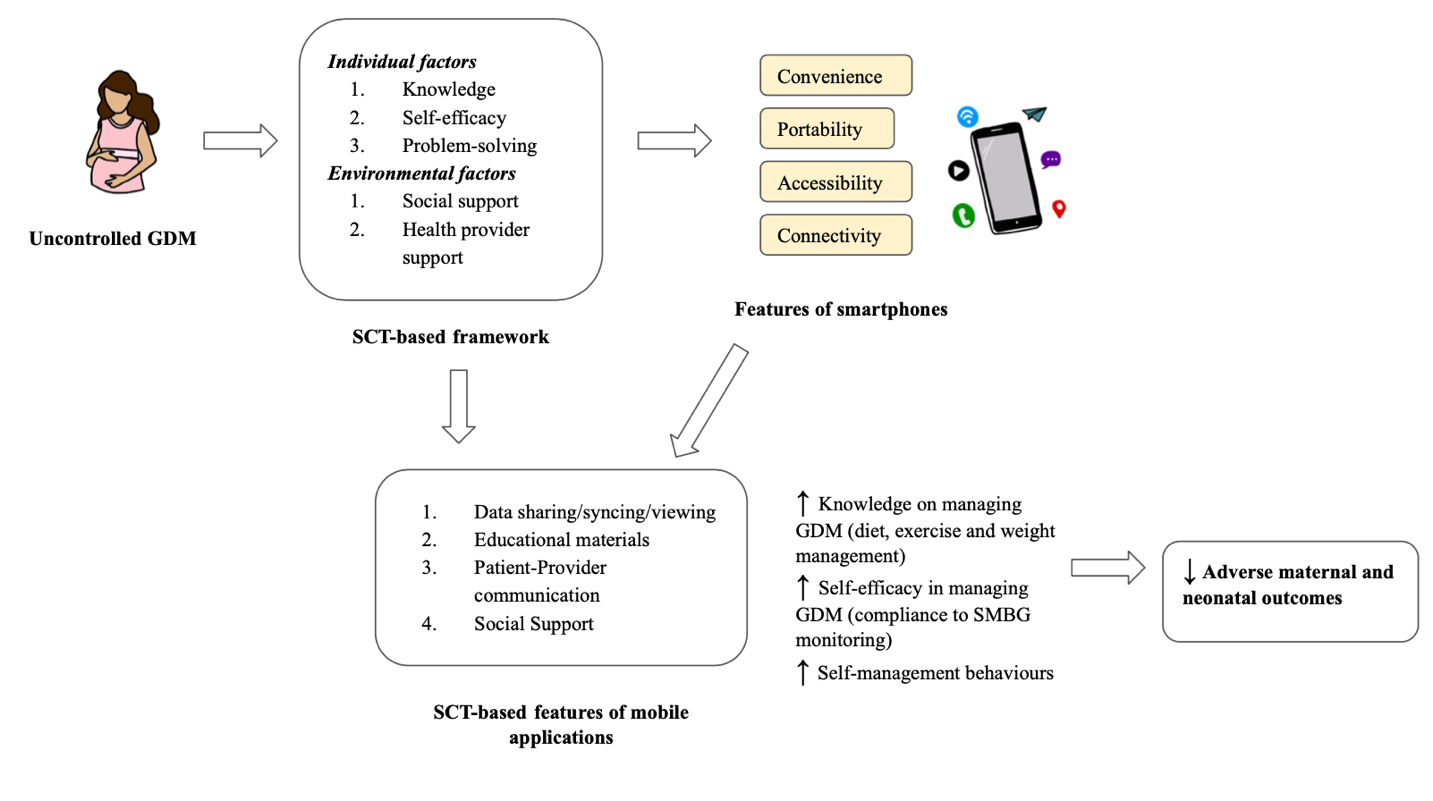
**
