# Supplementary material for: The Effect of Smartphone Application–Based Self-Management Interventions Compared to Face-to-Face Diabetic Interventions for Pregnant Women With Gestational Diabetes Mellitus: A Meta-Analysis
Source: J Diabetes Res. 2025 Mar 1;2025:4422330. doi: 10.1155/jdr/4422330 (PMC11986943; doi:10.1155/jdr/4422330)
Supplement: Supporting Information 3 — Eligibility criteria. [file 4422330.f3.docx]

**The effect of smartphone application-based self-management interventions compared to face-to-face diabetic interventions for pregnant women with gestational diabetes mellitus: A meta-analysis**

Supporting Information 3: Eligibility criteria.

| Criteria | Inclusion Criteria | Exclusion Criteria |
| --- | --- | --- |
| Population | Pregnant women with gestational diabetes mellitus (GDM) according to any type of GDM diagnostic criteria (International Association of Diabetes and Pregnancy Study Group criteria (IADPSG) endorsed by the World Health Organization (WHO), the International Federation of Gynecology and Obstetrics (FIGO) criteria or the Carpenter-Coustan criteria. | - People with previous prior of Type 1 Diabetes Mellitus and Type 2 Diabetes Mellitus - Women with recently resolved GDM post-delivery |
| Intervention | Smartphone application-based interventions which facilitate self-management of GDM.  Features can include any one of the following:   - Data sharing/syncing/viewing - Educational materials - Patient-provider communication - Social support | - Smartphone application is only one component of an entire complex intervention. - Apps which only involve one-way communication from provider to participants |
| Comparison | Standard clinical care/usual care which involves face-to-face interventions, without the use of smartphones |  |
| Outcomes | Maternal outcomes:   - fasting plasma glucose (FPG) - 2-hour postprandial plasma glucose (2h-PPG) - haemoglobin A1c (HbA1c) - gestational weight gain - Incidence of C-section)   Neonatal outcomes:   - Birthweight - Incidence of macrosomia - Incidence of large for gestational age (LGA) - Incidence of hypoglycemia - Neonatal intensive care unit (NICU) admission |  |
| Study Designs | Randomized Controlled Trials | Non-experimental, quasi-experimental, qualitative designs |
| Publication Types | Full text published and unpublished | Abstracts, book chapter review, conference proceedings, editorial |
