# Supplementary material for: The Effect of Smartphone Application–Based Self-Management Interventions Compared to Face-to-Face Diabetic Interventions for Pregnant Women With Gestational Diabetes Mellitus: A Meta-Analysis
Source: J Diabetes Res. 2025 Mar 1;2025:4422330. doi: 10.1155/jdr/4422330 (PMC11986943; doi:10.1155/jdr/4422330)
Supplement: Supporting Information 4 — Search terms in databases. [file 4422330.f4.docx]

**The effect of smartphone application-based self-management interventions compared to face-to-face diabetic interventions for pregnant women with gestational diabetes mellitus: A meta-analysis**

Supporting Information 4: Search terms in databases.

| **Database** | **Search Strategy** |
| --- | --- |
| PubMed | "diabetes, gestational"[MeSH Terms] OR GDM[Title/Abstract] OR "gestational diabetes"[Title/Abstract] OR "gestational diabetes mellitus"[Title/Abstract] OR ("diabetes"[Title/Abstract] AND "pregnancy-induced"[Title/Abstract]) OR ("pregnancy"[Title/Abstract] AND "glucose intolerance"[Title/Abstract]) OR "pregnancy induced diabetes"[Title/Abstract] OR (“child bearing"[Title/Abstract] AND diabetes[Title/Abstract]) OR (women[Title/Abstract] AND diabetes[Title/Abstract])  AND  "Internet-Based Intervention"[MeSH Terms] OR "Mobile Applications"[MeSH Terms] OR "Social Media"[MeSH Terms] OR "mobile app*"[Title/Abstract] OR "phone app*"[Title/Abstract] OR "iphone app*"[Title/Abstract] OR "electronic app*"[Title/Abstract] OR "portable electronic app*"[Title/Abstract] OR "IOS"[Title/Abstract] OR "ios app*"[Title/Abstract] OR "android app*"[Title/Abstract] OR "android"[Title/Abstract] OR "software app*"[Title/Abstract] OR "smartphone app*"[Title/Abstract] OR WeChat[Title/Abstract] OR "Internet-Based Intervention"[Title/Abstract] OR "Social Media"[Title/Abstract] OR Digital[Title/Abstract]  AND  "randomized controlled trial"[Publication Type] OR random*[Title/Abstract] |
| EMBASE | “pregnancy diabetes mellitus”/exp OR “pregnancy diabetes mellitus”:ti,ab OR gdm:ti,ab OR “gestational diabetes":ti,ab OR “gestational diabetes mellitus”:ti,ab OR “pregnancy induced diabetes”:ti,ab OR ((wom* NEAR/2 diabetes):ti,ab) OR ((mother* NEAR/2 diabetes):ti,ab)  AND  “social media”/exp OR “web-based intervention”/exp OR “mobile application”/exp OR “online system”/exp OR “mobile app*”:ti,ab OR “phone app*”:ti,ab OR “iphone app*”:ti,ab OR “electronic app*”:ti,ab OR “portable electronic app*”:ti,ab OR ios :ti,ab OR “ios app*”:ti,ab OR “android app*”:ti,ab OR android:ti,ab OR “software app*”:ti,ab OR “smartphone app*”:ti,ab OR WeChat:ti,ab OR ((internet NEAR/1 base*):ti,ab) OR “online system”:ti,ab OR digital:ti,ab  AND  “crossover procedure”:de OR “double-blind procedure”:de OR “randomized controlled trial”:de OR “single-blind procedure”:de OR random*:de,ab,ti OR crossover*:de,ab,ti OR ((cross NEXT/1 over*):de,ab,ti) OR ((doubl* NEAR/1 blind*):de,ab,ti) OR ((singl* NEAR/1 blind*):de,ab,ti) OR assign*:de,ab,ti OR allocat*:de,ab,ti OR rct:ab,ti OR “randomly allocated”:ab,ti OR “allocated randomly”:ab,ti OR “random allocation”:ab,ti OR ((allocated NEAR/2 random):ab,ti) |
| CINAHL | MH “diabetes mellitus, gestational” OR TI ( "diabetes, gestational" OR gdm OR "gestational diabetes" OR "gestational diabetes mellitus" OR "diabetes, pregnancy" OR "diabetes, women" OR "glucose intolerance, pregnancy" OR "childbearing, diabetes" OR "pregnancy-induced diabetes" ) OR AB ( "diabetes, gestational" OR gdm OR "gestational diabetes" OR "gestational diabetes mellitus" OR "diabetes, pregnancy" OR "diabetes, women" OR "glucose intolerance, pregnancy" OR "childbearing, diabetes" OR "pregnancy-induced diabetes" )  AND  MH “mobile applications” OR MH “telehealth+” OR MH “internet-based intervention” OR TI ( “mobile app*” OR “phone app*” OR “iphone app*” OR “electronic app*” OR “portable electronic app*”OR ios OR “ios app*”OR “android app*” OR android OR “software app*” OR “smartphone app*” OR WeChat OR “online system” OR digital ) OR AB ( “mobile app*” OR “phone app*” OR “iphone app*” OR “electronic app*” OR “portable electronic app*”OR ios OR “ios app*”OR “android app*” OR android OR “software app*” OR “smartphone app*” OR WeChat OR “online system” OR digital )  MH "Randomized Controlled Trials+" OR MH “double-blind studies” OR MH “single-blind studies” OR MH “random assignment” OR TI (random* OR allocat* OR assign* OR control* OR rct OR crossover) OR AB (random* OR allocat* OR assign* OR control* OR rct OR crossover) OR PT (randomized controlled trial) |
| Scopus | (TITLE-ABS-KEY ( "diabetes, gestational"  OR  gdm OR  "gestational diabetes"  OR  "gestational diabetes mellitus"  OR  "diabetes, pregnancy"  OR  "diabetes, women"  OR  "glucose intolerance, pregnancy"  OR  "childbearing, diabetes"  OR  "pregnancy-induced diabetes" )  AND  TITLE-ABS-KEY ( "Mobile app*"  OR  "Internet-based intervention"  OR  "Social media"  OR  app OR application* OR  "phone app*"  OR  "portable electronic app*"  OR  IOS  OR  Wechat  OR  "smartphone app*"  OR  android  OR  "android app*" )  AND  TITLE-ABS-KEY ( "clinical trials"  OR  "randomized controlled trial"    OR  "random allocation"  OR  "randomly allocated"  OR  "allocated randomly"  OR  "double-blind method"  OR  "single-blind method"  OR  "cross-over Studies"  OR  "cross-over trial"  OR  "single blind"  OR  "double blind" ) |
| Cochrane Library | #1 MeSH descriptor: [Diabetes, Gestational] explode all trees 1175  #2 ("diabetes, gestational” OR gdm OR "gestational diabetes" OR "gestational diabetes mellitus" OR "diabetes, pregnancy" OR "diabetes, women" OR "glucose intolerance, pregnancy" OR "childbearing, diabetes" OR "pregnancy-induced diabetes"):ti,ab,kw 3359  #3 MeSH descriptor: [Mobile Applications] explode all trees 1144  #4 MeSH descriptor: [Internet-Based Intervention] explode all trees 377  #5 MeSH descriptor: [Digital Technology] explode all trees 8  #6 MeSH descriptor: [Social Media] explode all trees 281  #7 ("Mobile app*” OR "Internet-based" OR "Social media" OR app* OR "phone app*" OR "portable electronic app*" OR IOS OR WeChat OR "smartphone app*" OR "android" OR "android app"):ti,ab,kw 14784  #8 #1 OR #2 3413  #9 #3 OR #4 OR #5 OR #6 OR #7 15164  #10 #8 AND #9 110 |
| Web of Science | TS=("diabetes, gestational" OR gdm OR "gestational diabetes" OR "gestational diabetes mellitus" OR "diabetes, pregnancy" OR "diabetes, women" OR "glucose intolerance, pregnancy" OR "childbearing, diabetes" OR "pregnancy-induced diabetes" )  AND  TS=( "Mobile app*" OR "Internet-based" OR "Social media" OR app OR application* OR "phone app*" OR "portable electronic app*" OR IOS OR WeChat OR "smartphone app*" OR android OR "android app" )  AND  TS=( "clinical trials" OR "randomized controlled trial" OR "random allocation" OR "randomly allocated" OR "allocated randomly" OR "double-blind method" OR "single-blind method" OR "cross-over studies" OR "cross-over trial" OR "single blind" OR "double blind" ) |
