# Supplementary material for: The Effect of Smartphone Application–Based Self-Management Interventions Compared to Face-to-Face Diabetic Interventions for Pregnant Women With Gestational Diabetes Mellitus: A Meta-Analysis
Source: J Diabetes Res. 2025 Mar 1;2025:4422330. doi: 10.1155/jdr/4422330 (PMC11986943; doi:10.1155/jdr/4422330)
Supplement: Supporting Information 5 — Excluded full-text articles with reasons (n = 26). [file 4422330.f5.docx]

**The effect of smartphone application-based self-management interventions compared to face-to-face diabetic interventions for pregnant women with gestational diabetes mellitus: A meta-analysis**

Supporting Information 5: Excluded full text articles with reasons (n = 26).

| Author, Year | Articles | Reasons |
| --- | --- | --- |
| Davis et al., 2018 | Optimizing gestational weight gain with the eating4two smartphone app: Protocol for a randomized controlled trial | Wrong population |
| Garnweidner-Holme et al., 2020 | Effect of the pregnant+ smartphone app on the dietary behavior of women with gestational diabetes mellitus: Secondary analysis of a randomized controlled trial | Not RCT |
| Ghaderi et al., 2021 | The effect of smartphone-based education on self-efficacy of women with gestational diabetes: A controlled clinical trial | Wrong outcome |
| Ghasemi et al., 2021 | Comparing the effect of individual counseling with counseling on social application on self-care and quality of life of women with gestational diabetes | Not RCT |
| Guo et al., 2021 | Application of online-offline integrated medical care management in patients with gestational diabetes | Wrong intervention |
| Hirst et al., 2015 | Acceptability and user satisfaction of a smartphone-based, interactive blood glucose management system in women with gestational diabetes mellitus | Not RCT |
| Hirst et al., 2016 | Digital blood glucose monitoring could provide new objective assessments of blood glucose control in women with gestational diabetes | Not RCT |
| Homko et al., 2007 | Use of an internet-based telemedicine system to manage underserved women with gestational diabetes mellitus | Wrong intervention |
| Huang et al., 2021 | Effect of mobile health based peripartum management of gestational diabetes mellitus on postpartum diabetes: A randomized controlled trial | Wrong outcome |
| Huhn et al., 2020 | Effectiveness of real-time continuous glucose monitoring to improve glycaemic control and pregnancy outcome in patients with gestational diabetes mellitus | Wrong intervention |
| Ji et al., 2020 | Impact of clinical pharmacist intervention on blood glucose control and perinatal outcomes in gestational diabetes mellitus through a diabetes management system | Wrong intervention |
| Kennelly et al., 2018 | Pregnancy exercise and nutrition with smartphone application support: A randomized controlled trial | Wrong population |
| Kim et al., 2012 | Development of an internet-based glucose management system for glucose control of pregnant women | Wrong intervention |
| Loerup et al., 2013 | GDM-health: Telehealth for remote monitoring and treatment of gestational diabetes | Not RCT |
| Loerup et al., 2015 | A comparison of blood glucose metrics to assess the feasibility of a digital health system for management of women with gestational diabetes: The GDM-health study | Not RCT |
| Mackillop et al., 2014 | Development of a real-time smartphone solution for the management of women with or at high risk of gestational diabetes | Not RCT |
| Moazen et al, 2021 | The effectiveness of telemedical monitoring program diabcare tirol for patients with gestational diabetes mellitus | Not RCT |
| Potzel at al., 2022 | A novel smartphone app to change risk behaviors of women after gestational diabetes: a randomized controlled trial | Wrong population |
| Poulter et al., 2022 | Use of a smartphone-based, interactive blood glucose management system in women with gestational diabetes mellitus: A pilot study | Not RCT |
| Rasekaba et al., 2018 | Using technology to support care in gestational diabetes mellitus: Quantitative outcomes of an exploratory randomised control trial of adjunct telemedicine for gestational diabetes mellitus (TeleGDM) | Wrong intervention |
| Su et al., 2021 | Effectiveness of a nurse-led web-based health management in preventing women with gestational diabetes from developing metabolic syndrome | Wrong population |
| Tumminia et al., 2019 | "Mysweetgestation": A novel smartphone application for women with or at risk of diabetes during pregnancy | Not RCT |
| Yee et al., 2022 | A feasibility randomized trial of a smartphone application for low-income pregnant individuals with diabetes | Wrong population |
| Zeng et al, 2023 | Information-based continuous nursing on pregnant women with gestational diabetes mellitus | Wrong intervention |
| Zhang at al. 2019 | Effectiveness of low glycemic index diet consultations through a diet glycemic assessment app tool on maternal and neonatal insulin resistance: A randomized controlled trial | Wrong population |
| Zhang et al., 2021 | The effects of the instantaneous scanning glucose monitoring system on hypoglycemia, weight gain, and health behaviors in patients with gestational diabetes: A randomised trial | Wrong intervention |
