# Supplementary material for: The Effect of Smartphone Application–Based Self-Management Interventions Compared to Face-to-Face Diabetic Interventions for Pregnant Women With Gestational Diabetes Mellitus: A Meta-Analysis
Source: J Diabetes Res. 2025 Mar 1;2025:4422330. doi: 10.1155/jdr/4422330 (PMC11986943; doi:10.1155/jdr/4422330)
Supplement: Supporting Information 6 — Exclusion of trials from registries with reasons (n = 19). [file 4422330.f6.docx]

**The effect of smartphone application-based self-management interventions compared to face-to-face diabetic interventions for pregnant women with gestational diabetes mellitus: A meta-analysis**

Supporting Information 6: Exclusion of trials from registries with reasons (*n* = 19).

| ID | Article | Reason |
| --- | --- | --- |
| ClinicalTrials.gov (*n* = 14) | | |
| NCT03324737 | Smartphone app to restore optimal weight in women with recent gestational diabetes (SPAROW) | Wrong population |
| NCT05489536 | Optimizing gestational weight gain for prevention of gestational diabetes mellitus in Malaysia | Wrong population |
| NCT05597943 | Mhealth smartphone app and postpartum glucose intolerance for patients with GDM | Ongoing/ Not retrievable |
| NCT05348863 | A digital platform to improve self-management of gestational diabetes (SPARK) | Ongoing/Not retrievable |
| NCT03504592 | Smartphone utilization for glucose monitoring and antenatal reporting (SUGAR) | Wrong population |
| NCT05521893 | Effectiveness of telemedicine care replacing standard care in gestational diabetes | Not retrievable |
| NCT03491436 | Pregnancy remote monitoring of women at risk for gestational diabetes | Withdrawn/Not retrievable |
| NCT04198857 | Development and testing of a mobile health application for management of gestational diabetes | Ongoing/Not retrievable |
| NCT05204706 | The Malaysian gestational diabetes and prevention of diabetes study (MyGODDESS) | Ongoing/Not retrievable |
| NCT05003154 | Digitalized management exploration for gestational diabetes mellitus in China | Ongoing/Not retrievable |
| NCT03987412 | A behavioral intervention to prevent gestational diabetes mellitus (DIGITAL-G) | Wrong population |
| NCT03340311 | Evaluating the feasibility of using m-health to improve serum glucose logs | Not RCT |
| NCT03669887 | Lifestyle modification to improve diet in women with GDM | Wrong outcome |
| NCT03987438 | A behavioral intervention to prevent impaired glucose tolerance diabetes mellitus (DIGITAL-I) | Wrong population |
| WHO International Clinical Trials Registry Platform (ICTRP) (n=5) | | |
| ACTRN12617000169347 | Efficacy of the eating4two smartphone application for the prevention of excessive gestational weight gain: A randomised controlled trial. | Not retrievable |
| ChiCTR2000038031 | Evaluation of integrated intervention model of gestational diabetes mellitus based on medical internet of things (mIoT) | Not retrievable |
| ChiCTR2100049194 | Prospective randomized controlled study of the impact of Internet based patient education on pregnancy outcome in gestational diabetes mellitus (GDM) | Ongoing/Not retrievable |
| CTRI/2022/05/042382 | Smartphone app for management of gestational diabetes mellitus in urban and rural settings in India: A randomized controlled trial | Ongoing/Not retrievable |
| DRKS00022923 | Randomized controlled trial to evaluate the digital health application mysugr pro | Wrong population |
| IRCT20220909055918N1 | Reviewing the effect of self-care education through social media on self-care of overweight and obese pregnant women | Wrong population |
