# Supplementary material for: The Effect of Smartphone Application–Based Self-Management Interventions Compared to Face-to-Face Diabetic Interventions for Pregnant Women With Gestational Diabetes Mellitus: A Meta-Analysis
Source: J Diabetes Res. 2025 Mar 1;2025:4422330. doi: 10.1155/jdr/4422330 (PMC11986943; doi:10.1155/jdr/4422330)
Supplement: Supporting Information 7 — Excluded articles from other sources with reasons (n = 10). [file 4422330.f7.docx]

**The effect of smartphone application-based self-management interventions compared to face-to-face diabetic interventions for pregnant women with gestational diabetes mellitus: A meta-analysis**

Supporting Information 7: Excluded articles from other sources with reasons (*n* = 10).

| Author, Year | Article | Reason |
| --- | --- | --- |
| Google Scholar (n=2) | | |
| Manoharan et al., 2022 | Effectiveness of mobile call reminders and health information booklet to improve postnatal blood glucose monitoring among mothers with gestational diabetes mellitus receiving care from a tertiary health centre, Puducherry - A randomized controlled trial | Wrong intervention |
| Minschart et al., 2020 | Mobile-based lifestyle intervention in women with glucose intolerance after gestational diabetes mellitus (MELINDA), a multicenter randomized controlled trial: methodology and design | Wrong population |
| Citation Searching (n=8) | | |
| Bartholomew et al., 2015 | Managing diabetes in pregnancy using cell phone/internet technology | Wrong population |
| Cheung et al., 2019 | A pilot randomised controlled trial of a text messaging intervention with customisation using linked data from wireless wearable activity monitors to improve risk factors following gestational diabetes | Wrong population |
| Cui et al., 2019 | Value of short message service on blood glucose control and prognosis in patients with gestational diabetes | Not retrievable |
| Jiang et al., 2016 | The effect of WeChat follow-up on compliance behavior of pregnant women with gestational diabetes mellitus | Not retrievable |
| Kennelly et al., 2018 | Pregnancy exercise and nutrition with smartphone application support | Wrong population |
| Lim et al., 2021 | A smartphone app to restore optimal weight (SPAROW) in women with recent gestational diabetes mellitus: Randomized controlled trial | Wrong population |
| Yang et al., 2018 | Medical nutrition treatment of women with gestational diabetes mellitus by a telemedicine system based on smartphones | Not RCT |
| Zhao et al., 2018 | Effects of mobile home care platform on blood glucose and quality of life in gestational diabetes mellitus patients | Not retrievable |
