# Supplementary material for: The Effect of Smartphone Application–Based Self-Management Interventions Compared to Face-to-Face Diabetic Interventions for Pregnant Women With Gestational Diabetes Mellitus: A Meta-Analysis
Source: J Diabetes Res. 2025 Mar 1;2025:4422330. doi: 10.1155/jdr/4422330 (PMC11986943; doi:10.1155/jdr/4422330)
Supplement: Supporting Information 8 — Description of smartphone-based self-management interventions among included studies. [file 4422330.f8.docx]

**The effect of smartphone application-based self-management interventions compared to face-to-face diabetic interventions for pregnant women with gestational diabetes mellitus: A meta-analysis**

Supporting Information 8: Description of smartphone-based self-management interventions among included studies.

| Author, year | Definition of GDM | Intervention | Frequency | Duration | Care Provider |
| --- | --- | --- | --- | --- | --- |
| Al-ofi et al., 2018 | International Association of Diabetes and Pregnancy Study Group (IADPSG) | A Smartphone- Glucometer and a Glucomail application which includes  (i) Emergency alert given to doctor for hyperglycaemia or hypoglycaemia when threshold level was met  (ii) Gestational weight gain target is estimated by the application using the online calculator to calculate the weight gain per week to follow the guidelines from the Institute of Medicine for healthy weight gain during pregnancy  (iii) Proactive communication between healthcare professionals and patients through regular questionaries regarding GDM-related issues  (iv) Automated message from application to coach according to each pregnant women’s due date | BGM: 4 times daily (1 FPG, 3 2h PPG)  Body weight: every week | Diagnosis with GDM (24–28 weeks of gestation) and continued until 6 weeks post-delivery. (18-20 weeks throughout pregnancy and 6 weeks post-delivery) | Diabetic care team at GDM clinic |
| Borgen et al., 2019 | Non-specific criteria | Pregnant+ app which includes  (i) automatic/manual transfer of glucose values from glucometer, visualised on an interface which allows easy interpretation of results  (ii) examples of recommended physical activity with goal setting components  (iii) information of healthy diet with culturally-adapted food recommendations  (iv) general information on GDM-related issues | NR | From recruitment to 3 months postpartum | - |
| Guo et al., 2018 | ADA | DNurse app which includes  (i) automatic/manual data upload for review by doctor  (ii) online education by education nurse at an assigned time to answer questions regarding GDM-related issues such as diet, exercise, SMBG and insulin injection  (iii) notification on abnormal blood glucose result and analysis of possible underlying cause  (iv) general information on GDM-related issues | Online education available every night for 2 hours (if keen) | From recruitment to 3 months postpartum | Outpatient service doctor and education nurse |
| Huang et al., 2021 | International Federation of Gynaecology and Obstetrics (FIGO) | Wechat group which includes  (i) sharing of meals, exercise, records of blood glucose control and weight gain which is then supplemented with real-time guidance on self-management of GDM  (ii) peer interactions and support groups among the group members  (iii) general information on GDM-related issues  (iv) peer-learning and learning from multi-disciplinary professionals | Educational materials posted on Mondays and peer interaction throughout the week | From recruitment to delivery | researchers involved had been trained by a multidisciplinary management team consisting of obstetrics, endocrinologists, nutritionists, sports medicine experts, and health managers to ensure the consistency of management |
| Mackillop et al., 2018 | IADPSG | GDm-health app which includes  (i)alerted when threshold of blood glucose readings is reached  (ii)automated alert if participant is not recording a predefine ed number of glucose readings  (iii) text message which contains advice on diet, medication dose adjustments and encouragements sent periodically between clinic visits | NR | `From recruitment until delivery | Diabetes midwife |
| Maleki et al, 2023 | World Health Organization 2013 criteria | The educational app which includes  (i) information about GDM, nutrition, physical posture, exercise  (ii) ways of and recommendations for increasing spiritual growth, improving  interpersonal relationships, increasing responsibility and stress management  (iii) ways of contact with the researcher and software designer  (iv) recommendations  (v) other related programmes | NR | 4 weeks after intervention | researcher |
| Miremberg et al., 2018 | ADA | Glucosebuddy app which includes  (i) documentation of BG measurements  (ii) individualised feedback from clinical team on the participant’s glycaemic control, reassurance and positive messaging, dietary advice, insulin modification, alerts for scheduling of appointments  (iii) platform for communication between participants and clinic team regarding GDM management | Daily report | 12 months | maternal-fetal medicine specialist and endocrinologist |
| Munda et al, 2023 | IADPSG | Application which includes  (i) sending glucose readings  (ii) monthly individual video calls replacing on-site visits | Blood glucose profile monitoring daily / video call monthly | `From recruitment until delivery | Nurse and researcher |
| Perez-Ferre et al., 2010 | Carpenter-Coustan criteria | Mobile application which includes  (i)transmission of capillary glucose to central database via SMS  (ii) infrared transmission of glucose values stored in glucometer to cellular phone  (iii) glycaemic trend  (iv) feedback from health professionals regarding nutritional changes or insulin dose adjustments through text messages | NR | Recruitment until delivery | Endocrinologist, diabetes nurse educator |
| Simsek-Cetinkaya & Koc, 2022 | ADA | Smartphone app which includes  (i)digital education booklet about GDM (in different versions with audio, visual, and video)  (ii)diet compliance tracking (weight gain, number of meals, blood glucose levels, status of initiation of insulin therapy, and their insulin doses)  (iii)physical activity monitoring (type, intensity, and duration of their physical activity)  (iv)a platform to ask questions and receive immediate answers regarding any aspect of GDM management and synchronized and asynchronous nursing counselling  (v)Daily report on glucose, diet and physical activity status and SMS reminder for lack of data transmission  (vi)Online individual and group counselling | Online group counselling (once a week, 7 times in total, half an hour for each session) | 14 weeks | Nurses and researcher |
| Sung et al., 2019 | Non-specific criteria | Mobile phone application which includes  (i)automatic transfer of data from glucometer  (ii)regular messages once a week on recommendation for diet and exercise  (iii)communication with health care providers  (iv)tailored medical and nutritional guidance | Dietary and physical activity related messages once a week | Recruitment until delivery | Endocrinologist, nurses and nutritionists |
| Tian et al., 2021 | IADPSG | Wechat group which allows for  (i)active management of GDM which includes diet advice, examples of meals from other group members, and exercise rules  (ii) sharing of photos of their meals and snacks, daily exercise, and experience regarding BG control among the group members  (iii) usage of personalized guidance and also from the experiences of other group members  (iv) education on GDM management  (v) peer interactions and support groups | Briefing to encourage participation on Mondays; Sharing of lessons and articles on weekends | Recruitment until delivery | Researchers who had been uniformly trained |
| Yew et al., 2020 | World Health Organization 2013 criteria | Habits GDM app which includes  (i)prompting of SMBG and automatic transfer of data from glucometer to the app  (ii) a database of common food in Singapore is incorporated into the app and automated messages from app to record their diet  (iii) Bluetooth weighing scale which automatically upload weight onto the app and automated messaged to remind participants to weight weekly; this also provides an optimal GWG  (iv) manual chat function to interact with the health care team | 7 point capillary SMBG 2 days a week  Weight measurement reminder weekly | Recruitment until delivery | Diabetic nurse educator, dietician, obstetrician |
| Zhou and Gan, 2023 | ADA | Using video and the WeChat public platform to regularly impart knowledge and behaviour education to women with GDM and supervise the implementation of their actions. | NR | Recruitment until delivery | obstetricians, dietitians, psychological consultants and nursing staff |
| Zhuo et al., 2022 | ADA | Continuing Medical Care (CMC) app which includes  (i)manual upload of SMBG values, insulin dose, diet and exercise  (ii) educational articles and videos mainly concerning insulin storage information, IIT, treatment for injection phobia, and measures to cope with insulin injection omissions  (iii) real-time, interactive platform between patients and pharmacists on insulin management  (iv) Online active follow-up from pharmacists initiated when ongoing hyper- or hypoglycaemia was recognized through alerts | 4 point capillary SMBG 3 days a week | Recruitment until 12 weeks postpartum | Diabetic clinical pharmacists |
