# Supplementary material for: The Effect of Smartphone Application–Based Self-Management Interventions Compared to Face-to-Face Diabetic Interventions for Pregnant Women With Gestational Diabetes Mellitus: A Meta-Analysis
Source: J Diabetes Res. 2025 Mar 1;2025:4422330. doi: 10.1155/jdr/4422330 (PMC11986943; doi:10.1155/jdr/4422330)
Supplement: Supporting Information 9 — Features of smartphone application–based self-management interventions. [file 4422330.f9.docx]

**The effect of smartphone application-based self-management interventions compared to face-to-face diabetic interventions for pregnant women with gestational diabetes mellitus: A meta-analysis**

Supporting Information 9: Features of smartphone application-based self-management interventions.

| Author, Year | Data sharing/ syncing/ viewing | Educational materials | Patient-provider communication | Social support |
| --- | --- | --- | --- | --- |
| Al-ofi et al., 2018 | √ | √ | √ |  |
| Borgen et al., 2019 | √ | √ |  |  |
| Guo et al., 2018 | √ | √ | √ |  |
| Huang et al., 2021 |  | √ | √ | √ |
| Mackillop et al., 2018 | √ | √ |  |  |
| Maleki et al, 2023 |  | √ |  |  |
| Miremberg et al., 2018 | √ | √ | √ |  |
| Munda et al, 2023 | √ | √ |  |  |
| Pérez-Ferre et al., 2010 | √ | √ |  |  |
| Simsek-Cetinkaya & Koc, 2022 | √ | √ | √ | √ |
| Sung et al., 2019 | √ | √ | √ |  |
| Tian et al., 2021 |  | √ | √ | √ |
| Yew et al., 2021 | √ | √ | √ |  |
| Zhou and Gan, 2023 |  | v | √ |  |
| Zhuo et al., 2022 | √ | √ | √ |  |
