# Supplementary material for: The Effect of Smartphone Application–Based Self-Management Interventions Compared to Face-to-Face Diabetic Interventions for Pregnant Women With Gestational Diabetes Mellitus: A Meta-Analysis
Source: J Diabetes Res. 2025 Mar 1;2025:4422330. doi: 10.1155/jdr/4422330 (PMC11986943; doi:10.1155/jdr/4422330)
Supplement: Supporting Information 10 — Risk of bias Version 2 summary. [file 4422330.f10.docx]

**The effect of smartphone application-based self-management interventions compared to face-to-face diabetic interventions for pregnant women with gestational diabetes mellitus: A meta-analysis**

Supporting Information 10: Risk of Bias Version 2 Summary

**Intention-to-treat**

| **Study ID** | **D1** | **D2** | **D3** | **D4** | **D5** | **Overall** |
| --- | --- | --- | --- | --- | --- | --- |
| Borgen et al., 2019 |  |  |  |  |  |  |
| Mackillop et al., 2018 |  |  |  |  |  |  |
| Munda et al, 2023 |  |  |  |  |  |  |
| Sung et al., 2019 |  |  |  |  |  |  |
| Yew et al., 2021 |  |  |  |  |  |  |
| Zhuo et al., 2022 |  |  |  |  |  |  |
| Zhou and Gan, 2023 |  |  |  |  |  |  |

**Per-protocol**

| **Study ID** | **D1** | **D2** | **D3** | **D4** | **D5** | **Overall** |
| --- | --- | --- | --- | --- | --- | --- |
| Al-ofi et al., 2018 |  |  |  |  |  |  |
| Guo et al., 2018 |  |  |  |  |  |  |
| Huang et al., 2021 |  |  |  |  |  |  |
| Maleki et al, 2023 |  |  |  |  |  |  |
| Miremberg et al., 2018 |  |  |  |  |  |  |
| Perez-Ferre et al., 2010 |  |  |  |  |  |  |
| Simsek-Cetinkaya & Koc, 2022 |  |  |  |  |  |  |
| Tian et al., 2021 |  |  |  |  |  |  |

| D1 | Randomisation process | \|  \| \| --- \| | Low risk |
| --- | --- | --- | --- | --- |
| D2 | Deviations from the intended interventions | \|  \| \| --- \| | Some concerns |
| D3 | Missing outcome data | \|  \| \| --- \| | High risk |
| D4 | Measurement of the outcome |  |  |
| D5 | Selection of the reported result |  |  |
