# Supplementary material for: The Effect of Smartphone Application–Based Self-Management Interventions Compared to Face-to-Face Diabetic Interventions for Pregnant Women With Gestational Diabetes Mellitus: A Meta-Analysis
Source: J Diabetes Res. 2025 Mar 1;2025:4422330. doi: 10.1155/jdr/4422330 (PMC11986943; doi:10.1155/jdr/4422330)
Supplement: Supporting Information 11 — Forest plot of effect sizes (Hedges' g) for maternal outcomes between smartphone application–based self-management intervention and face-to-face intervention groups. [file 4422330.f11.docx]

**The effect of smartphone application-based self-management interventions compared to face-to-face diabetic interventions for pregnant women with gestational diabetes mellitus: A meta-analysis**

Supporting Information 11: Forest plot of effect sizes (Hedges’g) for maternal outcomes

between smartphone application-based self-management intervention and face-to-face intervention groups

1. **Fasting plasma glucose**

**
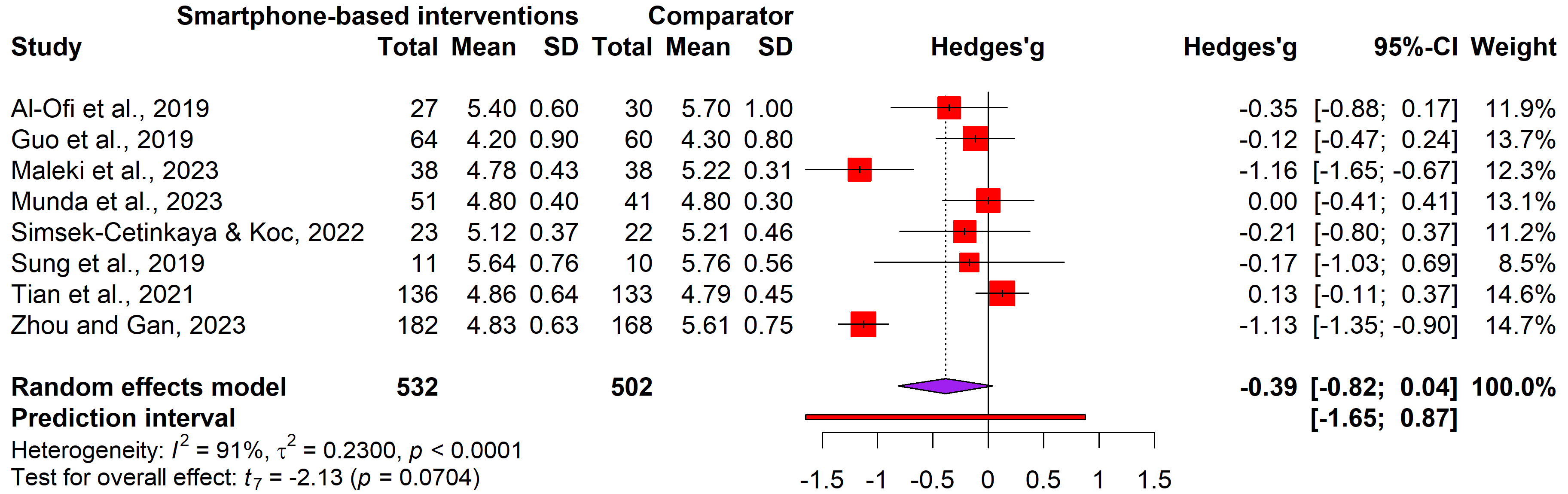
**

1. **2-hour postprandial plasma glucose**

**
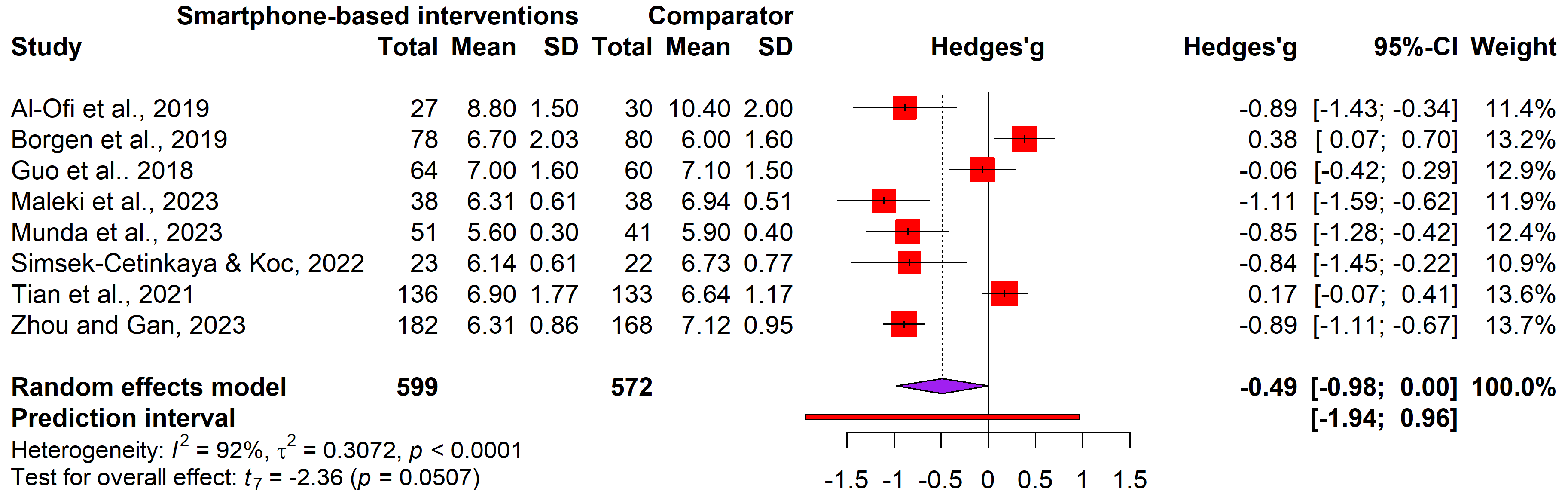
**

1. **HbA1c**

**
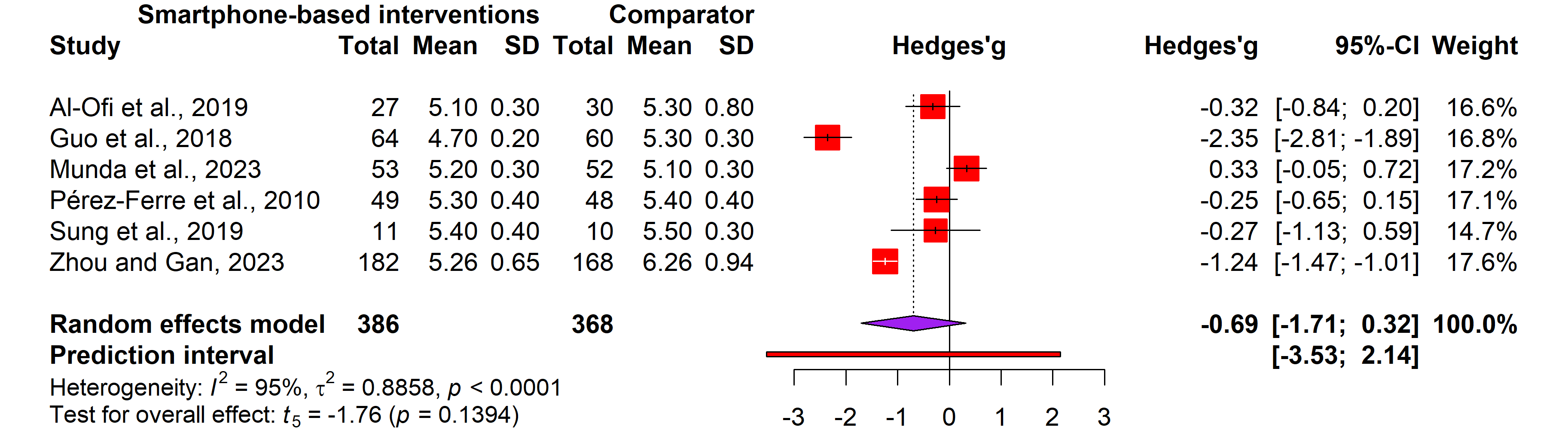
**

1. **Gestational weight gain**

**
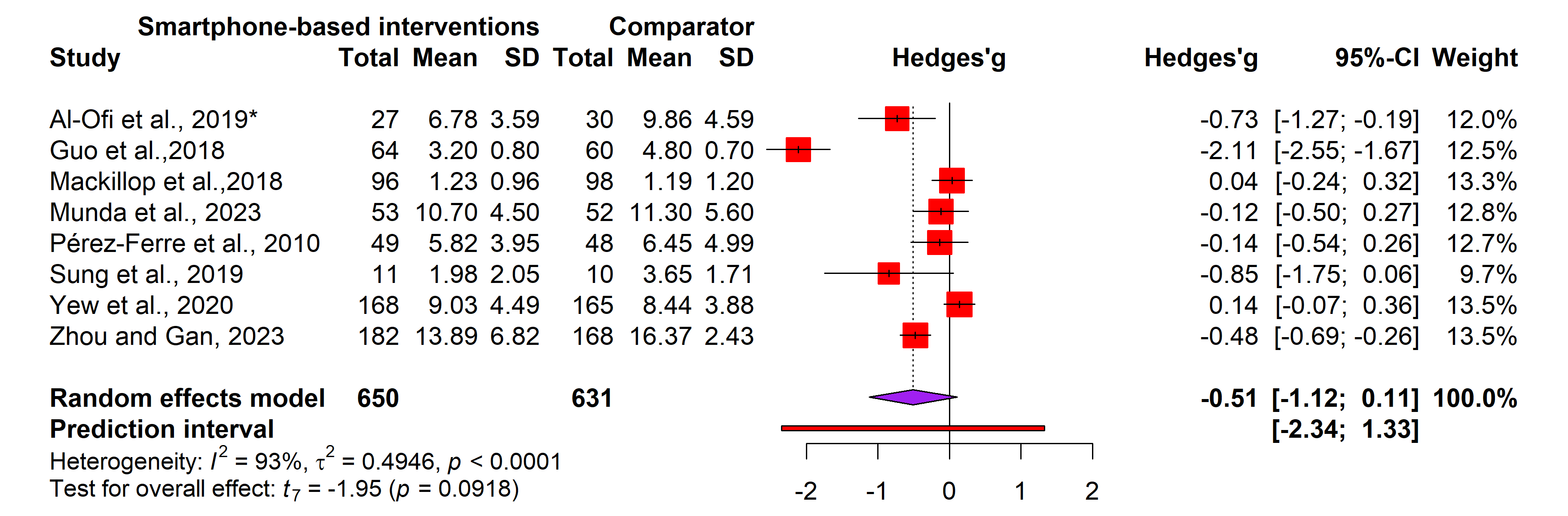
**
