# Supplementary material for: The Effect of Smartphone Application–Based Self-Management Interventions Compared to Face-to-Face Diabetic Interventions for Pregnant Women With Gestational Diabetes Mellitus: A Meta-Analysis
Source: J Diabetes Res. 2025 Mar 1;2025:4422330. doi: 10.1155/jdr/4422330 (PMC11986943; doi:10.1155/jdr/4422330)
Supplement: Supporting Information 13 — GRADE assessment for maternal and neonatal outcomes. [file 4422330.f13.docx]

**The effect of smartphone application-based self-management interventions compared to face-to-face diabetic interventions for pregnant women with gestational diabetes mellitus: A meta-analysis**

Supporting Information 13: GRADE assessment for maternal and neonatal outcomes.

| Certainty assessment | | | | | | | № of patients | | Effect | | Certainty | Importance |
| --- | --- | --- | --- | --- | --- | --- | --- | --- | --- | --- | --- | --- |
| № of studies | Study design | Risk of bias | Inconsistency | Indirectness | Imprecision | Other considerations | Smartphone-based self-management interventions | Face-to-face intervention | Relative (95% CI) | Hedges’ g (95% CI) |  |  |
| Fasting plasma glucose (mmol/L) | | | | | | | | | | | | |
| 8 | randomised trials | serious^a^ | very serious^b^ | serious^c^ | not serious | none | 532 | 502 | - | -0.39 (-0.82 – 0.04) | ⨁◯◯◯ Very low | IMPORTANT |
| 2-hour postprandial plasma glucose (mmol/L) | | | | | | | | | | | | |
| 8 | randomised trials | serious^a^ | very serious^b^ | serious^c^ | not serious | none | 599 | 572 | - | -0.49 (-0.98 – 0.00) | ⨁◯◯◯ Very low | IMPORTANT |
| HbA1c (%) | | | | | | | | | | | | |
| 6 | randomised trials | serious^a^ | very serious^b^ | serious^c^ | not serious | none | 386 | 368 | - | -0.69 (-1.71 – 0.32) | ⨁◯◯◯ Very low | IMPORTANT |
| Gestational weight gain (kg) | | | | | | | | | | | | |
| 8 | randomised trials | serious^a^ | very serious^b^ | serious^c^ | not serious | none | 650 | 631 | - | -0.51 (-1.12 – 0.11) | ⨁◯◯◯ Very low | IMPORTANT |
| C-section delivery (Incidence) | | | | | | | | | | | | |
| 12 | randomised trials | serious^a^ | serious^b^ | serious^c^ | not serious | none | 398/1133 (35.1%) | 447/1132 (39.5%) | RR 0.88 (0.72 to 1.09) | - | ⨁◯◯◯ Very low | IMPORTANT |
| Neonatal birthweight (g) | | | | | | | | | | | | |
| 7 | randomised trials | not serious | not serious | serious^c^ | not serious | none | 499 | 496 | - | - <0.01 (-0.13 – 0.13) | ⨁⨁⨁◯ Moderate | IMPORTANT |
| Macrosomia (Incidence) | | | | | | | | | | | | |
| 6 | randomised trials | serious^a^ | not serious | serious^c^ | not serious | none | 46/679 (6.8%) | 64/694 (9.2%) | RR 0.76 (0.61 to 0.94) | - | ⨁⨁◯◯ Low | IMPORTANT |
| Large for gestational age (Incidence) | | | | | | | | | | | | |
| 6 | randomised trials | not serious | very serious^d^ | serious^c^ | not serious | none | 58/453(12.8%) | 66/435 (15.2%) | RR 0.92 (0.46 to 1.81) | - | ⨁⨁◯◯ Low | IMPORTANT |
| Neonatal hypoglycaemia | | | | | | | | | | | | |
| 7 | randomised trials | not serious | serious^b^ | serious^c^ | not serious | none | 64/677 (11.7%) | 103/655(15.7%) | RR 0.81 (0.45 to 1.43) | - | ⨁⨁◯◯ Low | IMPORTANT |
| NICU admission (Incidence) | | | | | | | | | | | | |
| 6 | randomised trials | not serious | not serious | serious^c^ | not serious | none | 65/643 (10.1%) | 97/657 (14.8%) | RR 0.69 (0.42 to 1.04) | - | ⨁⨁⨁◯ Moderate | IMPORTANT |
