# Supplementary material for: The Effect of Smartphone Application–Based Self-Management Interventions Compared to Face-to-Face Diabetic Interventions for Pregnant Women With Gestational Diabetes Mellitus: A Meta-Analysis
Source: J Diabetes Res. 2025 Mar 1;2025:4422330. doi: 10.1155/jdr/4422330 (PMC11986943; doi:10.1155/jdr/4422330)
Supplement: Supporting Information 14 — Funnel plot showing symmetrical distribution and Egger's test of 12 trials for caesarian delivery. [file 4422330.f14.docx]

**The effect of smartphone application-based self-management interventions compared to face-to-face diabetic interventions for pregnant women with gestational diabetes mellitus: A meta-analysis**

Supporting Information 14: Funnel plot showing symmetrical distribution and Egger’s test of 12 trials for caesarian delivery.

**
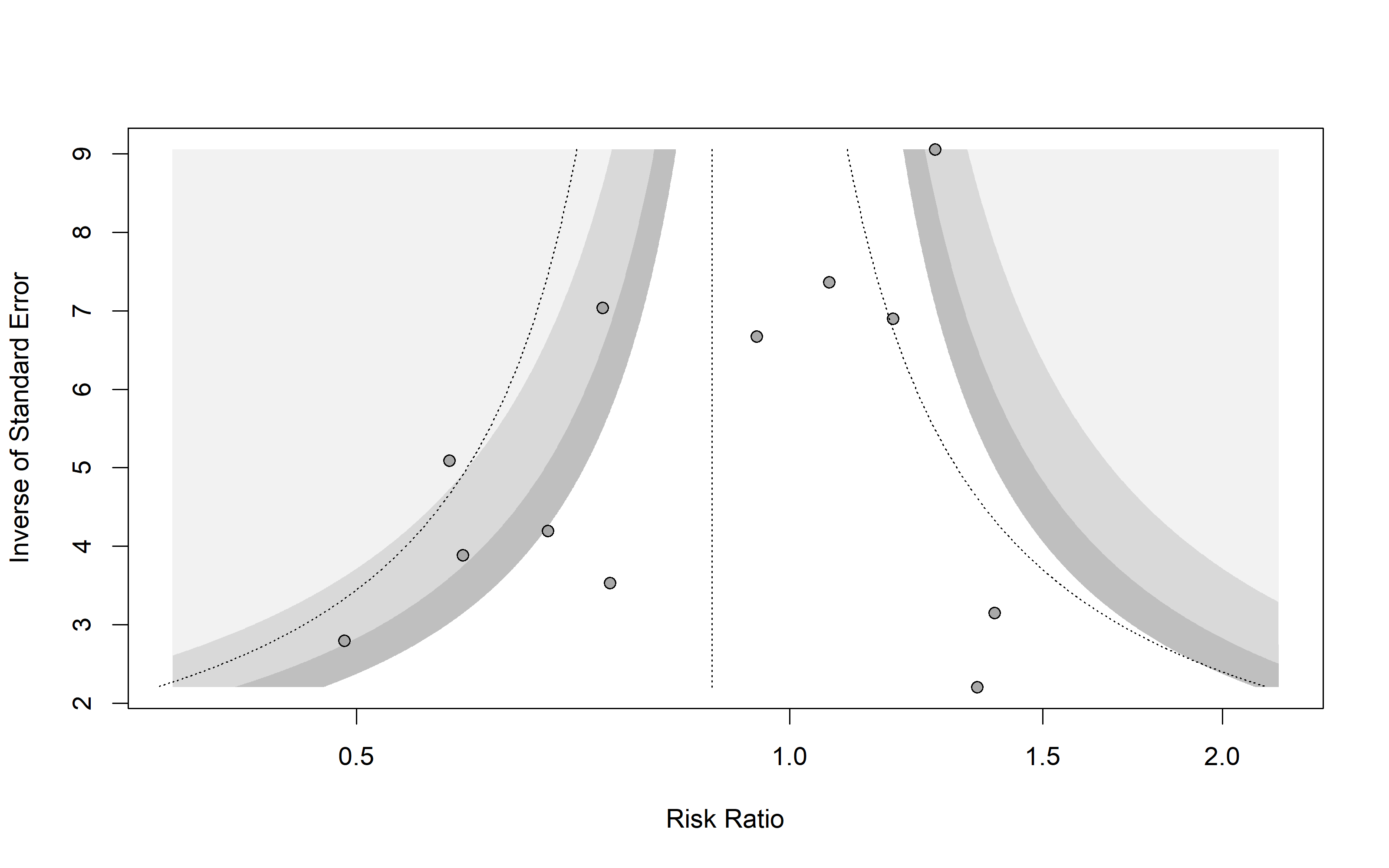
**

Linear regression test of funnel plot asymmetry

Test result: t = -1.54, df = 10, p-value = 0.1540

Sample estimates:

bias se.bias intercept se.intercept

-1.8574 1.2043 0.2554 0.2165

Details:

- multiplicative residual heterogeneity variance (tau^2 = 2.4562)

- predictor: standard error

- weight: inverse variance

- reference: Egger et al. (1997), BMJ
